# Evaluation of Viral RNA Recovery Methods in Vectors by Metagenomic Sequencing

**DOI:** 10.3390/v12050562

**Published:** 2020-05-19

**Authors:** Joyce Odeke Akello, Stephen L. Leib, Olivier Engler, Christian Beuret

**Affiliations:** 1 Biology Division, Spiez Laboratory, Swiss Federal Office for Civil Protection, Austrasse, CH-3700 Spiez, Switzerland; Olivier.Engler@babs.admin.ch; 2 Institute for Infectious Diseases, University of Bern, Friedbühlstrasse 51, 3001 Bern, Switzerland; stephen.leib@ifik.unibe.ch; 3 Graduate School for Cellular and Biomedical Sciences, University of Bern, Hochschulstrasse 4, 3012 Bern, Switzerland

**Keywords:** vector-borne viruses, vectors, sample processing, NGS, metagenomics

## Abstract

Identification and characterization of viral genomes in vectors including ticks and mosquitoes positive for pathogens of great public health concern using metagenomic next generation sequencing (mNGS) has challenges. One such challenge is the ability to efficiently recover viral RNA which is typically dependent on sample processing. We evaluated the quantitative effect of six different extraction methods in recovering viral RNA in vectors using negative tick homogenates spiked with serial dilutions of tick-borne encephalitis virus (TBEV) and surrogate Langat virus (LGTV). Evaluation was performed using qPCR and mNGS. Sensitivity and proof of concept of optimal method was tested using naturally positive TBEV tick homogenates and positive dengue, chikungunya, and Zika virus mosquito homogenates. The amount of observed viral genome copies, percentage of mapped reads, and genome coverage varied among different extractions methods. The developed Method 5 gave a 120.8-, 46-, 2.5-, 22.4-, and 9.9-fold increase in the number of viral reads mapping to the expected pathogen in comparison to Method 1, 2, 3, 4, and 6, respectively. Our developed Method 5 termed ROVIV (Recovery of Viruses in Vectors) greatly improved viral RNA recovery and identification in vectors using mNGS. Therefore, it may be a more sensitive method for use in arbovirus surveillance.

## 1. Introduction

Vectors transmit various viral infectious diseases of great public health concern [1]. Ticks and mosquitoes are known to be the most important vectors. Ticks transmit emerging viruses such as the tick-borne encephalitis virus (TBEV) which can cause serious infections [2,3], and mosquitoes transmit emerging arboviruses like dengue (DENV), chikungunya (CHIKV), and Zika (ZIKV) which also cause serious infections [4,5,6]. Identification and characterization of these viral genomes is widely based on sequencing methods to determine diversity of the virus and the epidemiological relationship between isolates within the population [7,8,9]. With mounting evidence of unbiased identification of pathogens, metagenomic next generation sequencing (mNGS) is a powerful tool to strengthen surveillance and rapidly respond to emerging viral vector-borne pathogens. Strengthening the identification and characterization of viral pathogens in vectors is thus vital in ensuring epidemic control.

A key aspect that may affect the identification of viral pathogens in vectors positive for emerging arboviruses using mNGS is the viral nucleic acid (NA) extraction. In recent years, great efforts have been made to develop extraction methods and evaluate their effectiveness in recovering NA from a wide range of biological and environmental samples [10,11,12]. A great majority of studies evaluating and comparing different extraction methods have dealt with selected sample types such as stool, tissue, serum, sewage, soil, and plants [13,14,15,16,17]. Studies evaluating different extraction methods for viral RNA sequence recovery in homogenized arthropods are lacking. One single extraction method may be applied to a wide range of sample types. However, it is important to select an extraction method suitable for the sample type being investigated, particularly when undertaking mNGS studies. This ensures that the maximum viral NA recovery is achieved by decreasing the amount of contaminants, thus avoiding unnecessary background noise that can interfere with mNGS analysis.

**Figure 1:**
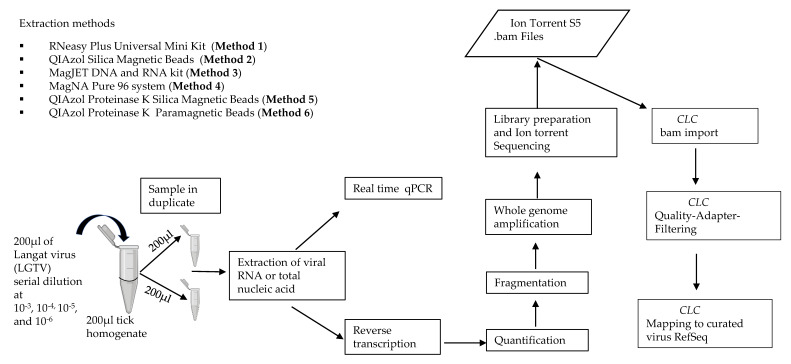
Schematic representation of the experiment design used for evaluating extraction methods for viral vector-borne metagenomic next generation sequencing (mNGS) analysis.

Due to the lack of studies evaluating extraction methods for viral RNA recovery in vectors by mNGS, our study aimed to evaluate the performance of six different NA extraction methods and systematically study their effects using homogenized arthropods. Evaluation of NA extraction methods was performed by exploring total RNA extraction, and total NA extraction based on different chemistries including silica column, silica magnetic beads, and glass particle magnetic beads. Evaluation was performed using various concentrations of TBEV surrogate Langat virus (LGTV), spiked into known negative tick (*Ixodes ricinus*) homogenates. The data showed variation in the performance of the different extraction methods as evaluated by qPCR and mNGS. This led to the development of a novel RNA extraction protocol that greatly improved viral RNA recovery and identification in homogenized arthropods. The sensitivity of the developed method was tested using naturally known TBEV positive tick (*Ixodes ricinus*) homogenates. As a proof of concept, the developed method was applied to experimentally infected DENV-2, CHIKV, and ZIKV positive mosquito (*Aedes aegypti*) homogenates. Herein, we developed an efficient and reproducible end-to-end sample processing pipeline for identification of viruses in vectors using mNGS (Figure 1, Supplementary File 1).

Overall, we demonstrated that the selected extraction method for viral RNA recovery in homogenized arthropods determines the reliability of qPCR and mNGS results. We also present our developed viral RNA extraction method ROVIV (Recovery of Viruses in Vectors) that may be suitable for use in arbovirus surveillance. This method is applicable to any RNA metaviromics protocol, including the identification and characterization of viruses in any biological or environmental sample. To our knowledge, this is the first study to evaluate different extraction methods for viruses in vectors using mNGS.

## 2. Materials and Methods

### 2.1. LGTV Viral Stock and Serial Dillutions

A stock solution of TBEV surrogate LGTV (strain TP12) grown in vero cells (African green monkey kidney epithelial cells) was serially diluted in ten-fold using sterile phosphate buffered saline ((PBS w/o CaCl_2_ & MgCl_2_, sterile-filtered, Merck KGaA) from 10^−1^ to 10^−9^ (viral culture supernatant of 5.3 × 10^7^ PFU/mL). Ten aliquots of each serial dilution at a volume of 200 µL in DNA LoBind 1.5 mL tubes (Eppendorf AG) were stored at −80 °C until further use. Duplicates of each 200 µL serial dilution were then extracted using the RNeasy Plus Universal Mini Kit known as Method 1 in this study. This extraction method was chosen because it is one of the most common commercial kits used for extraction of total viral RNA. The extracts were subjected to qPCR to determine Ct values and thus estimate LGTV genome copies for each serial dilution before undertaking spike sample preparation.

### 2.2. LGTV Spike Sample Prepartaion

Subsamples of a pool of five adult tick homogenates identified as negative for TBEV in a previous surveillance study [18] stored at −80 °C were thawed, centrifuged at 500 *g* for 3 min and spiked with serial dilutions of LGTV. Ten-fold serial dilutions of LGTV at 10^−3^, 10^−4^, 10^−5^, and 10^−6^ were chosen for the spiking experiments to mimic moderate-to-low viral loads that may be present in vectors. Briefly, 200 µL of each LGTV serial dilution at 10^−3^, 10^−4^, 10^−5^, and 10^−6^ was spiked into 200 µL of known negative tick homogenate and mixed well by vortexing. The mixture was centrifuged briefly then split into two tubes of 200 µL sample each and extracted as duplicates using the different extraction methods (Figure 1). This ensured that all samples representing each LGTV serial dilution contained roughly the same amount of spiked virus and homogenate.

### 2.3. Viral RNA Extraction Methods

**Figure 2:**
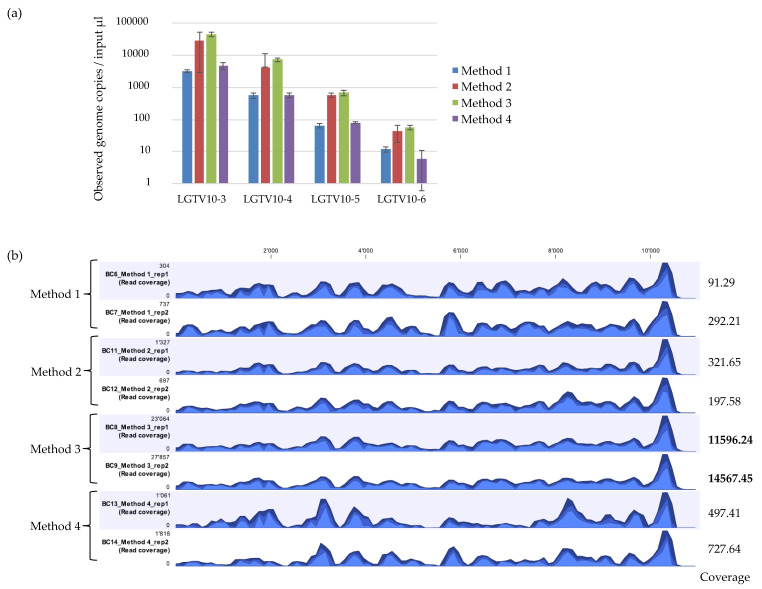
(**a**) Real time qPCR of observed genome copies recovered from Method 1, Method 2, Method 3, and Method 4. Reactions were performed in duplicates on duplicate extractions from LGTV serial dilutions at 10^−3^, 10^−4^, 10^−5^, and 10^−6^ spiked into tick homogenates. Error bars represent means ± SD. N = 4. (**b**) Coverage track profiles of LGTV mapped reads to the reference genome (TP21 EU790644) recovered using Method 1, 2, 3, and 4. The y-axis on the left within the coverage tracks indicated read coverage. Average coverage values are indicated for each sample on the right. The three blue shades show the minimum (light blue), mean (medium blue), and maximum (dark blue) observed values in a given region.

To extract and recover viral RNA from naturally negative adult tick homogenates (pools of 5 ticks) spiked with TBEV surrogate LGTV, four different NA extraction methods (Method 1, 2, 3, and 4) were first tested and evaluated (Table 1). For Method 1, 3, and 4, samples were extracted following the manufacturer’s instructions without the addition of carrier RNA. Method 2 is an in-house optimized method that follows the same procedure as Method 1 except that the silica column is replaced with silica magnetic beads (G-Bioscience, St Louis Missouri, USA). A further two NA extraction methods (Method 5 and 6) were later developed so as to assess the effectiveness of proteinase K and magnetic beads from two different suppliers (G-Bioscience, and ThermoFisher Scientific Inc., Reinach, Switzerland) on viral RNA recovery for mNGS analysis. The decision to assess the effectiveness of proteinase K and magnetic beads was due to the difference in the results observed (Figure 2a,b) for the extraction methods that did not contain proteinase K (Method 1, 2, and 4) and for methods that used magnetic beads from a different supplier (Method 2 and 3). Therefore, Method 5 and 6 which both included enzymatic digestion with proteinase K (ThermoFisher Scientific Inc.) during the lysis step and utilized silica magnetic beads (G-Bioscience) and paramagnetic beads (ThermoFisher Scientific Inc.) respectively for viral RNA capture were also tested and evaluated. A negative control consisting of PBS spiked into naturally negative tick homogenates was used to control for cross contamination in each extraction. The elution volume for all extraction methods was standardized to 50 µL.

### 2.4. Evaluating Performance of the Different Extraction Methods

To evaluate the efficiency of the different extraction methods, three approaches were used. These included (1) comparison of the recovered LGTV genome copies as determined by qPCR; (2) comparison of the recovered viral reads using mNGS analysis; and (3) a comparison of genome coverage profiles as determined by mapping recovered viral reads to the reference genome.

Following identification of the most efficient extraction method, the sensitivity and proof of concept of this extraction method was tested and evaluated. For testing sensitivity, naturally known TBEV positive tick (*Ixodes ricinus*) homogenates in a pool of five adult ticks from a previous surveillance study were used [19]. In addition, a subset of mosquito (*Aedes aegypti* RecLab Strain, Brazil) samples in pools of 8, raised in the FIOCRUZ insectary, Recife, Brazil and fed with a blood mixture containing either DENV-2 or ZIKV and/or CHIKV were also tested to demonstrate application (proof of concept) of the optimal method to other vector samples. The extracts of TBEV, DENV-2, CHKIV, and ZIKV were detected using real-time PCR (see real-time PCR) and subsequently subjected to mNGS.

### 2.5. qPCR

For estimation of the recovered copy number of spiked LGTV in negative tick homogenates from each sample by the different extraction methods, extracts were assessed using qPCR. The LGTV primer systems used were a validated in-house primer-probe set targeting the NS3 gene—forward primer 5′-TGTGTGGAGCGGCGATT-3′, reverse primer 5′-TAAGGGCGCGTTCCATCTC-3′, and the TaqMan probe FAM-CTTGGCCCCCACACGAGTGGTG-BHQ-1. The qPCR analyses were performed on a LightCycler^®^ 96 Real-Time PCR System (Roche, Diagnostics International AG) using TaqMan Fast Virus 1-Step Master Mix (Applied Biosystems^TM^, ThermoFisher Scientific Inc.) Briefly, a volume of 5 µL viral NA extract was combined with 20 µL mastermix containing TaqMan Fast Virus 1-Step Master Mix (Applied Biosystems^TM^, ThermoFisher Scientific Inc.), 0.4 µM of probe, forward and reverse primers, and nuclease free water. The cycling conditions were: 50 °C for 300 s, 95  °C for 20 s, and 45 cycles of 95  °C for 3 s, and 60 °C for 30  s. qPCR was performed in duplicates on duplicate extractions. Estimation of the LGTV copy number was based on a Ct-value of 21.32 corresponding to a viral genome copy number of 1.0 × 10^5^ per input 5 µL (slope of −3.44 and intercept of 38.54/log). The fold-change in LGTV genome copies between the extraction methods was calculated by finding the difference between the LGTV genome copies of samples from Method 2, 3, or 4 and LGTV genome copies of Method 1 samples divided by the LGTV genome copies of Method 1.

### 2.6. Real-Time PCR

For detection of TBEV, DENV-2, CHIKV, and ZIKV, extracts were assessed using real-time PCR. The real-time PCR analyses were performed on a LightCycler^®^ 96 System (Roche Diagnostics International AG) using TaqMan^®^ Fast Virus 1-Step Master Mix for RNA analysis (Applied Biosystems™, Thermo Fisher Scientific Inc.) The cycling conditions were: 300 s at 50 °C, 20 s at 95 °C, 45 × (3 s at 95 °C, 30 s at 60 °C). Real-time PCR was performed in duplicates on single extractions. The primers and probes used for detection of viral RNA of the different viral pathogens are provided in the Supplementary File 2—Table S1

### 2.7. Whole Genome Amplification (WGA)

Prior to WGA, the extracted NA was reverse-transcribed to cDNA synthesis using the SuperScript IV First-Strand Synthesis System (ThermoFisher Scientific Inc.) following the manufacturer’s instructions, using 11 µL of the extract and 1 µL of random primer. The concentration of cDNA was measured using the Qubit dsDNA High Sensitivity Assay Kit on the Qubit 3.0 Fluorometer (Thermo Fisher Scientific Inc.) as per manufacturer’s protocol. A total of 5 ng in 50 µL of each cDNA sample was fragmented using a Covaris M220 system according to the 400 bp library size protocol and 1 ng of fragmented cDNA was used in WGA using SeqPlex Enhanced DNA Amplification kit (SEQXE) according to the manufactures’ protocol.

### 2.8. Sequencing on the Ion Torrent S5 and Analyses

Ion torrent sequencing on the S5 platform was performed in-house. Barcoded mNGS libraries were created according to the Ion Xpress^TM^ Plus and Ion Plus Library preparation. Library preparation was automatically performed on the AB Library Builder^TM^ System using the Ion Plus Fragment Library Kit (ThermoFisher Scientific Inc.) Following library generation, size selection was performed on the adapter-ligated libraries using 0.55X Agencourt AMPure XP reagent (Beckman Coulter Eurocenter S.A.) The concentration of the size selected libraries was then measured using the Qubit dsDNA High Sensitivity Assay Kit. The size distribution of the size selected libraries assessed with the Agilent High Sensitivity DNA Kit (Agilent Technologies, Inc.) using the Agilent 2100 Bioanalyzer system. The sequencing run templates were planned on the Torrent Suite Software version 5.8, libraries were diluted in E1 buffer, pooled, and loaded on the Ion 530^TM^ chip using the Ion Chef^TM^ Instrument (Thermo Fisher Scientific). Ion torrent sequencing of the loaded chip was performed with 850 flows on the Ion S5^TM^ System using Ion 530^TM^ (400 bp) chip kit.

Following sequencing, the output BAM file was imported to CLC Genomics Workbench (version 12.0.3). The raw sequence reads from each sample were subjected to quality trimming of the adapter and barcode sequences. Reads < 50 nucleotides in length and low quality (score < 20) reads were discarded during quality trimming and filtering. All reads were then assembled reference-based using “CLC—map reads to reference”. Parameters for reference-based assembly consisted of match score = 1, mismatch cost = 2, length fraction = 0.5, similarity fraction = 0.8, insertion cost = 3, and deletion cost = 3. Parameters for duplicate removal included maximum representation of minority sequence at 20.0%. The sequencing depth and coverage were visually inspected using the CLC read track tool. Kraken 2 [20] standard database (downloaded 05202020) was used to perform taxonomic sequence classification. Visualization of the kraken 2 report was performed using Pavian [21].

### 2.9. Statistical Analyses

The difference in the efficiency of LGTV genome copy recovery for each sample by the different extraction methods was calculated with one-way ANOVA. The analysis was performed using the statistical R environment (version 3.5.0).

## 3. Results

To evaluate differences in RNA extraction methods, serial dilutions of LGTV was used to spike negative tick homogenates. The spiked LGTV serial dilutions at 10^−3^, 10^−4^, 10^−5^, and 10^−6^ represented mean Ct values of 21.35, 24.34, 28.68, and 31.92 respectively and equating to approximately 98,500, 13,300, 731, and 83.7 copies per 5 µL respectively. For the initial evaluation of the extraction methods, the fold changes were calculated in reference to the standard control method (Method 1). This method was chosen as a standard reference because it is one of the most common commercial kits used for total RNA extraction.

### 3.1. LGTV Viral Recovery Using qPCR and NGS Analysis

The detected LGTV varied between the different extraction methods with mean Ct values ranging between 22.67 to 26.47 for 10^−3^, 25.76 to 29.1 for 10^−4^, 28.81 to 32.32 for 10^−5^, and 32.6 to 36.25 for 10^−6^. The mean genome copies per 5 µL reaction ranged between 3205 to 44,400 copies for 10^−3^, 556.0 to 7310.0 copies for 10^−4^, 64.6 to 673.0 copies for 10^−5^, and 5.9 to 55.6 copies for 10^−6^ (Figure 2a). Method 3 had the highest fold difference compared to Method 2 and 4 in reference to the standard control Method 1 (Table 2). The difference in the efficiency of LGTV genome copy recovery was statistically significant (F = 11.93, *p* = 0.00295) between Method 2, 3 and 4. A large variation was observed for input LGTV serial dilution of 10^−6^ with Method 4. Method 2 and 3 had lower Ct values and thus, more viral genome copies recovered at any input LGTV serial dilution spiked into known negative tick homogenates compared to Method 1 and 4 therefore, suggesting that Method 2 and 3 may be more sensitive than Method 1 and 4.

mNGS analysis was performed on duplicate extractions for LGTV serial dilution at 10^−3^ using each extraction method. Reads were mapped to the LGTV reference genome (TP21 EU790644), and the percentage of mapped viral reads obtained. The viral recovery efficiency of the four extraction methods were different (Table 3). Method 2 and 3 showed consistent percentage of recovered viral reads mapping to the reference genome whilst, Method 1 and 4 had inconsistent mNGS results. Method 3 showed the highest percentage of recovered viral reads mapping to the reference genome, whilst Method 1 and 2 showed the lowest percentage of recovered viral reads. Comparison of the genome coverage track profile of each sample using the different extraction methods indicated that the overall pattern of coverage was relatively similar across the methods (Figure 2b). However, the percentage coverage of LGTV mapped reads to the LGTV reference genome varied relatively across the methods. Method 3 had the highest percentage coverage (99.6% and 100.0%), followed by Method 2 (97.3% and 96.4%), then Method 1 (96.2% and 96.9%), and finally Method 4 (90.2% and 95.0%). Inspection of the sequencing coverage for each sample extracted using the different extraction methods indicated that some regions of the LGTV genome at the same positions in samples extracted by Method 1 and 4 had no reads mapping to the reference. This was not the case for samples extracted with Method 2 and 3. Although Method 3 proved to outperform other extraction methods, particularly for mNGS analysis, the kit was discontinued by the supplier. Attempts were therefore made to develop other extraction methods.

### 3.2. ROVIV—QIAzol Lysis Accompanied with Proteinase K and Silica Magnetic Beads Improves Viral RNA Recovery

Two new extraction methods were developed based on QIAzol lysis, proteinase K digestion and silica magnetic beads. To assess the performance of the two newly developed extraction approaches (Method 5 and 6), these methods were evaluated along aside Method 2 and 3. Method 2 and 3 were chosen for further evaluation with the two newly developed extraction approaches because the initial qPCR and mNGS results (Figure 2a,b) suggested that these methods were more sensitive compared to Method 1 and 4. LGTV serial dilution at 10^−3^ spiked into known negative tick homogenates was used for evaluating these methods by both qPCR and mNGS analysis performed on duplicates extractions. LGTV serial dilution at 10^−3^ spiked into PBS (to act as a standard diluent) and extracted using Method 3 was also evaluated as a standard reference for qPCR analysis.

**Figure 3:**
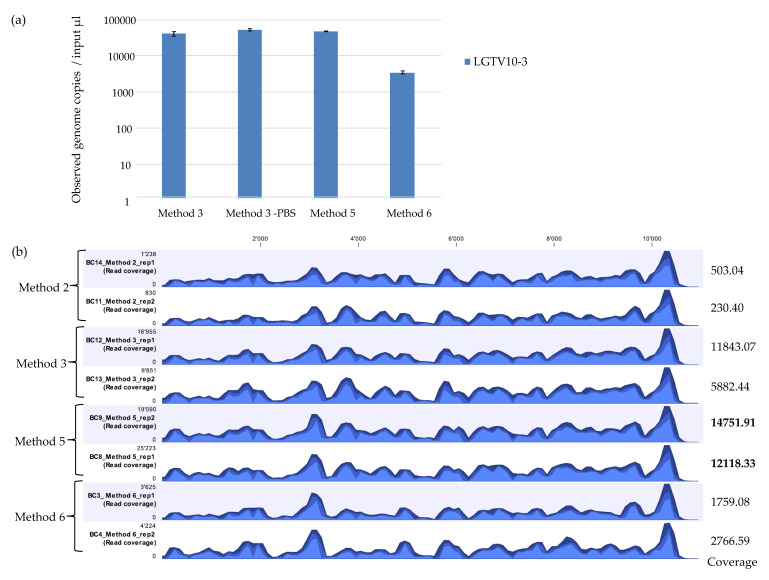
(**a**) Real time qPCR of observed genome copies recovered from further assessment of extraction methods (Method 3, Method 5, and Method 6). Reactions were performed in duplicates on duplicate extractions from LGTV serial dilution at 10^−3^ spiked into tick homogenates. Error bars represent means ± SD. N = 4. (**b**) Coverage track profiles of LGTV mapped reads to the reference genome (TP21 EU790644) recovered using Method 2, 3, 5, and 6. Average coverage values are indicated for each sample on the right. In the graph track, the three blue shades show the minimum (light blue), mean (medium blue), and maximum (dark blue) observed values in a given region.

qPCR demonstrated that Method 6 recovered the least amount of LGTV viral genome copies in comparison to Method 3 and Method 5 (Figure 3a). mNGS results showed that Method 2 recovered the least percentage (0.65% and 0.61%) of viral reads mapping to the LGTV reference genome. Method 6 recovered 3.03% and 3.05% LGTV viral reads. Method 3 recovered 11.94% and 11.54% LGTV viral reads. Method 5 showed the best viral recovery with percentage of 30.20% and 29. 45% LGTV viral reads (Table 4). This is 2.5 times the percentage of viral reads recovered by Method 3 which had proved to be the best method on initial assessment. Although Method 5 had the highest percentage of recovered viral reads and average coverage value, all the methods showed a highly similar pattern of genome coverage profile (Figure 3b). For the percentage coverage of LGTV mapped reads to the LGTV reference genome, Method 5 had a higher percentage coverage (99.0% and 99.1%) in comparison to Method 2, 3, and 6 which had 98.6% and 97.8%, 98.0% and 97.9%, and 98.0% and 97.7% coverage respectively. A method that achieves a high average coverage is desired to control errors in base calling and assembly. Overall, these results showed the outstanding performance of the newly developed Method 5 in comparison to Method 3 (Table 4), a method which had showed better performance on initial evaluation with other methods (Table 3, Figure 2a,b). Therefore, these results suggest that Method 5 may be more sensitive and desirable for use in vector mNGS than Method 1, 2, 3, 4, or 6.

### 3.3. Testing Sensitivity of the Optimized Method

**Figure 4:**
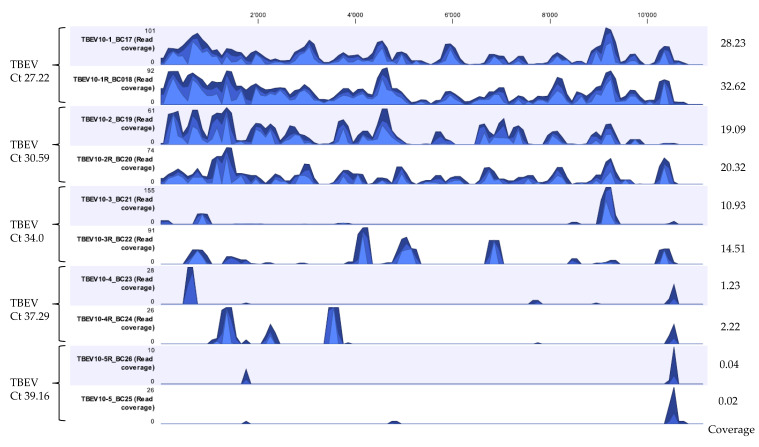
Coverage track profiles of TBEV showing the pattern of sequencing depth obtained for five different samples sequenced in duplicates with Ct values ranging from 27.22 to 39.16 extracted using the developed Method 5. Ion S5 reads from each sample were mapped on to the TBEV reference genome NC-001672.1. Average coverage values are indicated for each sample on the right. In the graph track, the three blue shades show the minimum (light blue), mean (medium blue), and maximum (dark blue) observed values in a given region.

After developing a suitable extraction method and establishing a workflow for viral vector-borne mNGS using LGTV spiked tick homogenate samples, the developed extraction Method 5—termed ROVIV—was tested for its sensitivity using naturally known TBEV positive tick homogenates. We processed and sequenced TBEV positive tick homogenates with Ct values ranging from 27.22 to 39.16. The moderate-to-high Ct value samples were chosen because of the difficulty in identifying viral pathogens from vectors when they are present in low quantities in comparison to the host background. Given the fact that our workflow did not include a host depletion step, TBEV viral sequencing reads for all the samples tested were efficiently recovered and identified (Table 5 and Figure 4). The percentage coverage of the TBEV mapped reads to the TBEV reference genome (NC-001672.1) was 88.3% and 94.7% for TBEV samples with Ct 27.22; 64.4% and 74.0% for TBEV sample with Ct 30.59; 15.0% and 30.0% for TBEV sample with Ct 34.0; 2.0% and 8.9% for TBEV sample with Ct 37.29; and 1.5% and 1.2% for TBEV sample with Ct 39.16. The coverage profile along with average coverage values for each TBEV sample are indicated in Figure 4.

**Figure 5:**
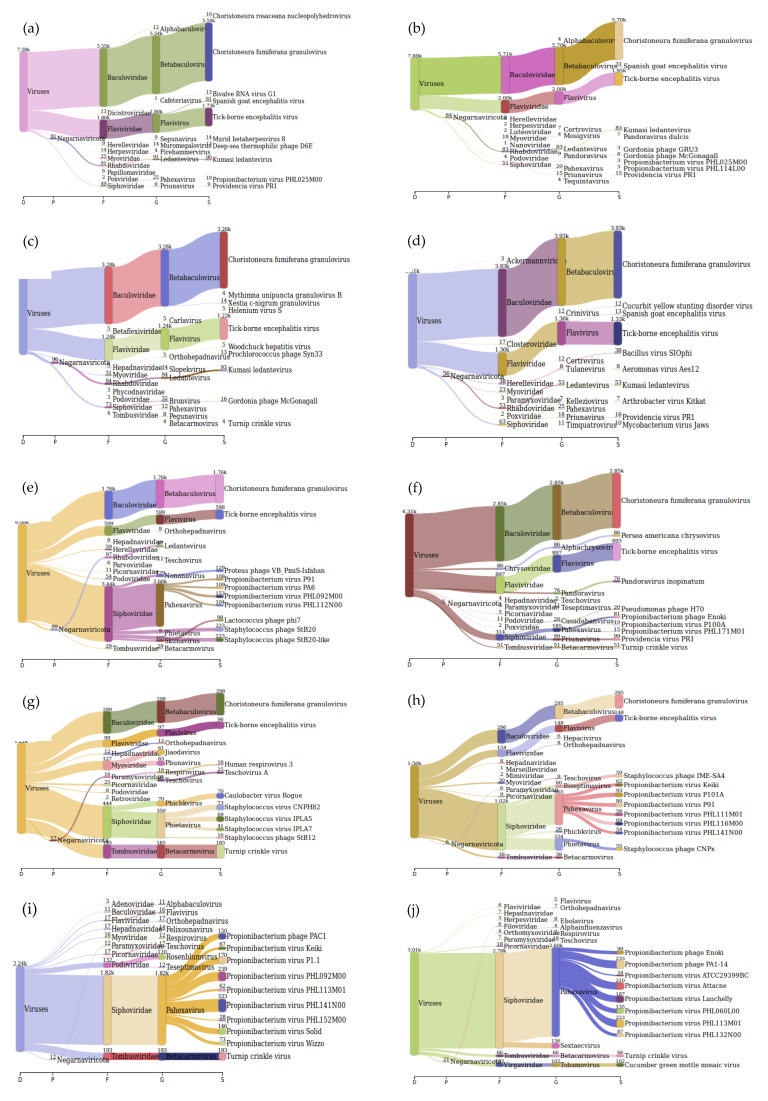
Sankey diagrams of Kraken 2 report results obtained for sensitivity of the sample preparation pipeline using tick-borne encephalitis virus (TBEV) positive tick homogenate. Panel (**a**,**b**) represent duplicate mNGS results of TBEV positive tick homogenate with Ct value 27.22, panel (**c**,**d**) of sample with Ct value 30.59, panel (**e**,**f**) of sample with Ct value 34.0, panel (**g**,**h**) of sample with Ct value 37.29, and panel (**i**,**j**) of sample with Ct value 39.16.

The Sankey interpretation of the kraken 2 report generated using Pavian [21] after processing for viruses only indicated that TBEV was identified in samples with Ct values from 27.22 to 37.29. For the sample with a Ct value of 39.16, TBEV was not identified. However, the family to which TBEV belongs to was identified (Figure 5).

### 3.4. Proof of Concept of the Optimized Method

**Figure 6:**
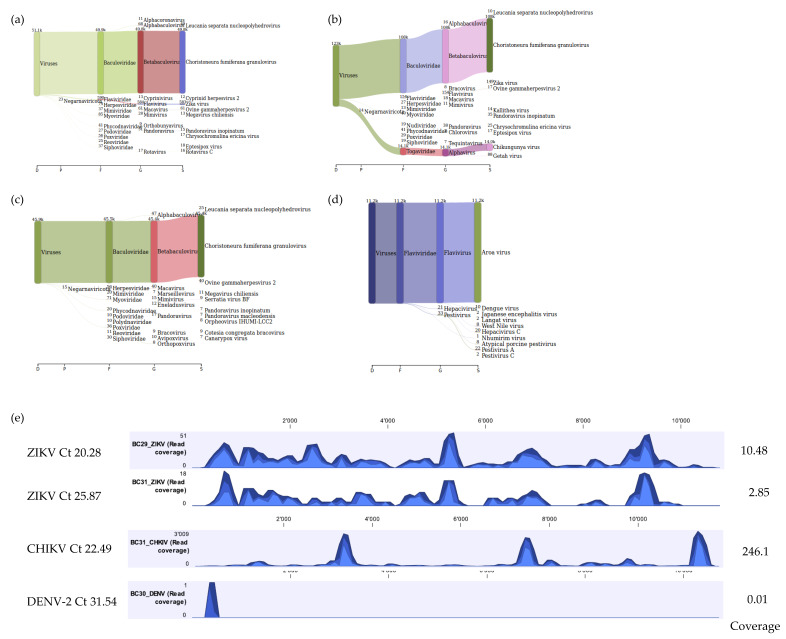
Sankey diagrams of Kraken 2 report results obtained for mosquito homogenates positive for ZIKV (panel **a**), ZIKV and CHIKV (panel **b**), and DENV-2 (panel **c**). Panel (**d**) represents results of DENV-2 using a custom database for flaviviruses only. Panel (**e**) demonstrates the coverage track profile of ZIKV, CHIKV, and DENV-2 samples extracted using the developed Method 5. Ion S5 reads for ZIKV were mapped on to the ZIKV reference genome (NC_012532.1), CHIKV mapped on to the CHIKV reference genome (NC_004162.2), and DENV-2 mapped onto the reference genome (NC_001474.2). Average coverage values are indicated for each sample on the right.

In addition, as a proof of concept for the use of our workflow for identification of viruses in vectors other than ticks, mosquito samples known to be positive for DENV-2, CHIKV, and ZIKV were tested. For the mosquito homogenate that was positive for both CHIKV and ZIKV (mean Ct values 22.49 and 25.87, respectively), a total of 5,028,470 reads were obtained with 14,289 (0.28%) and 146 (0.00%) viral reads mapping to CHIKV reference genome (NC_004162.2) and ZIKV reference genome (NC_012532.1) respectively. The CHIKV mapped reads covered approximately 98.9% of the CHIKV reference genome whilst the ZIKV mapped reads covered approximately 66.7% of the ZIKV reference genome. For the mosquito homogenate that was positive for ZIKV (mean Ct value 20.28), a total of 3,625,436 reads were obtained with 563 (0.02%) viral reads mapping to the ZIKV reference genome (NC_012532.1) covering approximately 92.8% of the ZIKV reference genome. mNGS analysis using Kraken 2 standard database also identified viral reads for ZIKV and CHIKV (Figure 6a,b). For the positive DENV-2 mosquito homogenate (mean Ct value 31.54), a total of 2,639,666 reads were obtained with 1 (0.00%) DENV-2 read recovered by mapping to reference genome (NC_001474.2). Although only one DENV-2 viral read mapped to the chosen DENV-2 reference genome and mNGS analysis using Kraken 2 standard database did not show any DENV reads, further mNGS analysis using a custom flavivirus database indicated that 10 DENV viral reads were present in the sample (Figure 6c,d). The coverage track profile for ZIKV, CHIKV, and DENV-2 is shown in Figure 6e.

## 4. Discussion

The increasing use of mNGS as a powerful tool to strengthen viral identification and characterization of known and novel emerging viruses in vectors [22,23,24,25] necessitates optimizing individual steps of the mNGS workflow. Successful recovery of viral NA in vectors including ticks and mosquitoes is challenging because they are covered with an exoskeleton of chitin that must be disrupted before the extraction process. Due to this, possible PCR and NGS inhibitors along with ribonucleases that can degrade NA are present. Evaluating and optimizing efficient viral NA extraction methods is thus crucial for mNGS analysis. Various commercial and non-commercial extraction methods have been used for recovery of NA from ticks and mosquitoes [26,27,28,29,30,31,32].

In this study, we evaluated the use of different extraction methods for their efficiency in recovering viral NA from vectors positive for viral pathogens of public health concern by subsequent mNGS analysis. We observed a substantial difference in the observed genome copies (Figure 2a and Figure 3a) and the percentage of recovered viral reads representing the expected pathogen by different extraction methods (Table 3 and Table 4). Interestingly, results from the mNGS analysis showed that the extraction methods evaluated in this study gave a different percentage of viral reads mapping to the expected pathogen despite having relatively similar qPCR results. This goes to show that qPCR results may not correlate with mNGS results. Moreover, qPCR results show that among the four initial extraction methods (Method 1–4) evaluated, Method 2 had a higher viral RNA recovery efficiency (lower Ct value) than Method 4 in recovery of LGTV viral NA (Ct values 22.98 vs. 25.63 respectively). However, the mNGS data showed that Method 4 recovered relatively more LGTV viral sequencing reads than Method 2. This result potentially hints that mNGS identification may not be merely dependent on Ct values. Differences in the coverage was also observed. To explain the possible lack of a relationship between qPCR and mNGS results, the coverage for the NS3 region that contain the PCR amplicon was calculated. For samples extracted using Method 2, 3, 5, and 6, the coverage for the NS3 region was approximately 100%. For Method 1, coverage was approximately 99.9% and for Method 4 the coverage was approximately 85.8% (for replicate 1) and 95.7% (for replicate 2). As Method 1 and Method 4 recovered the least LGTV copies (had relatively higher Ct value) compared to the other methods, the results for the coverage at the NS3 region containing the PCR amplicon possibly justifies the difference seen. Nevertheless, this does not seem to explain the possible reason why mNGS identification may not be merely dependent on the Ct values. Clearly, these observations confirm that the selected extraction method for viral NA recovery from vectors may determine the reliability of qPCR and mNGS results. In addition, it shows that not all viral NA extraction methods performing well for qPCR are better suited for viral vector-borne mNGS analysis. Optimization of viral NA extraction methods is thus a key aspect in viral vector-borne mNGS studies as the extraction method can impact the viral RNA yield, number/percentage of viral reads, and genome coverage, as well as robustness of the data.

A study comparing methods for genomic DNA extraction in mosquito larvae showed that the extraction method had a significant effect on the DNA yield [33]. Cruz-Ramos and colleagues showed a significant difference in the amount and purity of DNA obtained for two DNA extraction methods in larvae, pupae, and adult female *Aedes aegypti* [34]. These studies highlight the importance of comparing and evaluating different extraction methods for vectors depending on the research question at hand. Sathiamoorthy and colleagues showed that methods using precipitation techniques poorly recover single-stranded RNA viruses [35]. Extraction methods comprising viral NA precipitation were therefore not evaluated as the main focus was on recovering RNA viruses, particularly single-stranded RNA viruses. Single-stranded RNA viruses, e.g., *Coronaviridae* (SARS-CoV-2, SARS coronavirus), *Flaviviridae* (TBEV, dengue virus, zika virus), *Filoviridae* (Marburg virus, Ebola virus), *Hantaviridae* (orthohanta virus), *Paramyxoviridae* (Measles virus), and *Orthomyxoviridae* (Influenza A virus), are known to be the major relevant RNA viruses that cause a significant burden to human and animal health [36]. Therefore, utilizing methods that show better recovery of RNA viruses is crucial. Evaluation and optimization of sample processing methods thus depends on the sample type, expected pathogen, and the research question at hand. Viral NAs are usually present at low concentrations in vectors thus making selection and optimization of viral NA extraction methods difficult especially when trying to efficiently recover all viral NA present in the sample.

We developed an extraction method for viral vector-borne mNGS that maximizes the recovery of viral NA and gives outstanding mNGS results. On average, our developed extraction Method 5—termed ROVIV—gave 120.8-, 46-, 2.5-, 22.4-, and 9.9-fold increase in the percentage of viral reads mapping to the expected pathogen when compared with Method 1, 2, 3, 4, and 6, respectively. In addition, our method efficiently recovered TBEV viral sequencing reads from TBEV positive tick homogenates with Ct values ranging from 27.22 to 39.16 (Table 5 and Figure 4). DENV-2, CHIKV, and ZIKV viral reads from positive mosquito homogenates were also recovered. Although our workflow does not yet include tick and/or mosquito-specific ribosomal RNA depletion step, the results suggest that our method may be sensitive for identification of vector-borne viruses from vectors using mNGS even without a host depletion step. Nevertheless, including a host depletion step may greatly improve the sensitivity.

The ROVIV Method which consists of combining QIAzol lysis with enzymatic digestion by proteinase K followed by capture of viral NA with silica magnetic beads is efficient, reproducible, and gives a high yield of viral reads, and is thus suitable for mNGS analysis. Proteinase K, a nonspecific serine protease, is known to rapidly inactivate nucleases such as RNases and DNases during RNA and/or DNA extraction. Thus, in an event where there are traces of active RNases and DNases, this enzyme removes these nucleases, hence preventing degradation of the viral NA. Failure to add proteinase K may result in a reduced yield of viral NA. Silica columns and silica magnetic beads are widely used in extraction of NA. Use of silica column approach (Method 1) in this study resulted in less recovery of viral NA and viral reads mapping to the represented expected pathogen. This observation can be partially explained by the fact that cell debris from vector homogenates cause clogging of the silica column membrane, thus trapping viruses on the membrane and leading to insufficient elution of viral NA. Therefore, based on our study results, use of silica magnetic beads for viral NA capture in vectors is a better preferred option especially when a filtration step is not performed which was the case in this study. The amount of silica magnetic beads used in this study was 25 μL. He and colleagues demonstrated that 20 μL of silica magnetic beads are sufficient to guarantee a high recovery of NAs and also noted that the extraction efficiency reduced when a large amount of silica magnetic beads were added [37].

As the amount of viral load present in vectors is often low, it is crucial to perform amplification of the viral NA when conducting mNGS analysis so as to enrich for viral NAs. Studies utilizing random amplification for viral detection when mNGS analysis is applied have demonstrated its usefulness for low viral load samples despite the substantial bias associated with amplification [31,38]. Nevertheless, a second-strand synthesis approach to generate double-stranded DNA might be considered to avoid substantial bias, but may not be suitable for low viral load samples. In this study, we employed the use of the SeqPlex DNA amplification kit to facilitate enrichment of the viral NA. This kit utilizes primers composed of a universal 5’end and a semi-degenerate 3’end to perform amplification.

Wylezich et al. demonstrated that a versatile sample preparation method is beneficial for metagenomics [32]. Although our proposed method was designed and optimized for viral vectors including ticks and mosquitoes that are positive for emerging arboviruses (TBEV, DENV, CHIKV, and ZIKV), it can be applied to other environmental samples. Other vectors including rodents, fleas, midges, carrion flies, bed bugs, bat flies, *Culicoides*, and biological samples may yield optimal results with this approach. It would be interesting to investigate how well it performs when applied to a variety of different samples. Nevertheless, we did not have the opportunity to investigate this.

There are limitations to this study. Tick and/or mosquito-specific ribosomal RNA depletion was not included in our workflow for viral vector-borne mNGS and therefore, this may have impacted on the amount of virus recovered. Only one simplified bioinformatics approach was used and therefore, it might not have fully represented the number of viral reads recovered. One may therefore recover more viral reads if a different bioinformatic approach is used. Thus, combining our optimal extraction with optimal bioinformatics is ideal. Our developed extraction method is manual. Thus, in the case of large vector-borne epidemiological surveillance studies, it could be tedious and time consuming. Therefore, our extraction method would benefit more from being made automated.

Overall, our study demonstrated that the selected extraction method has a significant impact on mNGS analysis. The study therefore provides useful information to researchers studying viral vector-borne pathogens and those using new surveillance techniques such as Xenosurveillance and further helps with choice of RNA extraction method. Our proposed sample processing pipeline for viral vector-borne mNGS which is based on a developed extraction method termed ROVIV (Recovery of Viruses in Vectors), and WGA may be more sensitive and is suitable for use in the identification and characterization of known and novel viruses in vectors known to transmit pathogens of public health concern.

## Figures and Tables

**Table 1:** Extraction methods used in the study and reasons for their inclusion.

Extraction Method	Chemistry	Vendor/Supplier	Reason for Inclusion in the Study
RNeasy Universal Plus Mini Kit (Method 1)	Silica column	Qiagen	RNA extraction
QIAzol Silica Magnetic Beads (Method 2)	Silica magnetic beads	our lab-optimized protocol	RNA extraction, no clogging by cell debris
MagJET DNA and RNA Kit (Method 3)	Paramagnetic beads	ThermoFisher	Total nucleic acid extraction, no clogging by cell debris
MagNA Pure 96 System (Method 4)	Magnetic glass particles	Roche	Total nucleic acid extraction, high throughput
QIAzol Proteinase K Silica Magnetic Beads (Method 5)	Silica magnetic beads	our lab-optimized protocol	RNA extraction, no clogging by cell debris
QIAzol Proteinase K Paramagnetic beads (Method 6)	Paramagnetic beads	our lab-optimized protocol	RNA extraction, no clogging by cell debris

**Table 2:** The fold difference observed for the initial methods evaluated in reference to the standard control Method 1. LGTV = Langat virus.

Spiked Serial Dilution	Method 2	Method 3	Method 4
LGTV10^−3^	7.73	12.85	0.49
LGTV10^−4^	6.59	12.14	0.03
LGTV10^−5^	7.81	9.42	0.21
LGTV10^−6^	2.65	3.73	−0.49

**Table 3:** Recovered viral reads by mNGS analysis based on initial assessment of different extraction methods (Method 1, 2, 3, and 4) for metagenomic identification of viral vector-borne pathogens. Ct: cycle threshold. The best result is indicated in bold.

Extraction	Sample	Ct Value	Total Reads	Mapped Reads N (%)
Method 1	LGTV10^−3^ replicate 1	26.38	4,829,403	5367 (0.11%)
	LGTV10^−3^ replicate 2		4,528,711	17,536 (0.39%)
Method 2	LGTV10^−3^ replicate 1	22.98	6,876,209	20,016 (0.29%)
	LGTV10^−3^ replicate 2		4,398,740	11,821 (0.27%)
Method 3	LGTV10^−3^ replicate 1	**22.04**	3,588,781	**703,931 (19.61%)**
	LGTV10^−3^ replicate 2		4,426,097	**882,244 (19.93%)**
Method 4	LGTV10^−3^ replicate 1	25.63	3,748,017	31,925 (0.85%)
	LGTV10^−3^ replicate 2		2,526,069	46,636 (1.85%)

**Table 4:** Recovered viral reads by mNGS sequence analysis based on further assessment of extraction methods (Method 2, 3, 5, and 6) for metagenomic identification of viral vector-borne pathogens. Ct: cycle threshold. The best result is indicated in bold.

Extraction	Sample	Ct Value	Total Reads	Mapped Reads N (%)
Method 2	LGTV10^−3^ replicate 1	23.53	4,236,072	27,391 (0.65%)
	LGTV10^−3^ replicate 2		2,087,367	12,816 (0.61%)
Method 3	LGTV10^−3^ replicate 1	22.77	5,766,461	688,769 (11.94%)
	LGTV10^−3^ replicate 2		3,053,077	352,335 (11.54%)
Method 5	LGTV10^−3^ replicate 1	**22.72**	2,796,041	**844,300 (30.20%)**
	LGTV10^−3^ replicate 2		2,355,912	**693,898 (29.45%)**
Method 6	LGTV10^−3^ replicate 1	26.58	3,320,147	100,494 (3.03%)
	LGTV10^−3^ replicate 2		5,036,970	153,703 (3.05%)

**Table 5:** Output of mNGS results for TBEV positive tick homogenates used for testing sensitivity of the sample preparation pipeline. Samples with “R” at the end are replicates. Ct: cycle threshold.

Sample	Ct Value	Total Number Reads	% Classified Reads	% Viral Reads	% Bacterial Reads	% Chordate Reads	% Protozoan Reads	% Fungal Reads
TBEV10^−1^	27.22	3,607,592	47.4	0.21	13.4	32.2	0.0	0.0
TBEV10^−1^ R		3,465,868	38.3	0.228	11.1	25.3	0.0	0.0
TBEV10^−2^	30.59	3,198,211	34.8	0.148	10.9	22.4	0.0	0.0
TBEV10^−2^ R		4,262,864	35.1	0.129	9.0	24.6	0.0	0.0
TBEV10^−3^	34	3,155,979	58.9	0.193	31.5	26.3	0.0	0.0
TBEV10^−3^ R		2,852,515	48.8	0.151	25.4	21.9	0.0	0.0
TBEV10^−4^	37.29	3,222,131	70.3	0.038	43.5	26.4	0.0	0.0
TBEV10^−4^ R		3,082,738	70.9	0.0507	42.8	27.8	0.0	0.0
TBEV10^−5^	39.16	3,196,999	62.7	0.0701	26.5	35.9	0.0	0.0
TBEV10^−5^ R		2,572,154	49.4	0.117	39.6	9.32	0.0	0.0

## Supplementary Materials

The following are available online at https://www.mdpi.com/1999-4915/12/5/562/s1, Table S1: Primers and probes used for detection of viral pathogens in the real time qPCR assay, Supplementary file S1: Detailed description of the developed extraction method ROVIV and mNGS workflow protocol.
