# Supplementary material for: Evaluation of Viral RNA Recovery Methods in Vectors by Metagenomic Sequencing
Source: Viruses. 2020 May 19;12(5):562. doi: 10.3390/v12050562 (PMC7290855; doi:10.3390/v12050562)
Supplement: Supplementary file 1 [file viruses-12-00562-s001.pdf]

Supplementary file 1

Evaluation of Viral RNA Recovery Methods in Vectors by Metagenomic Sequencing

Running title of the supplementary file 1.
An efficient and reproducible end -to -end sample processing pipeline for viral vector-borne metagenomic next generation sequencing

Authors
Joyce Odeke Akello[1, 2, 3*], Stephen L. Leib[2], Olivier Engler[1], Christian Beuret[1*]
[1]Biology Division, Spiez Laboratory, Swiss Federal Office for Civil Protection, Austrasse, CH-3700 Spiez, Switzerland.
[2]Institute for Infectious Diseases, University of Bern, Friedbühlstrasse 51, 3001 Bern, Switzerland.
[3]Graduate School for Cellular and Biomedical Sciences, University of Bern, Hochschulstrasse 4, 3012 Bern, Switzerland.

[*]**Correspondence**

Dr. Christian Beuret, Spiez Laboratory, Swiss Federal Office for Civil Protection, Austrasse CH-3700, Spiez, Switzerland. Phone +41584681664, E-mail: Christian.Beuret@babs.admin.ch

Joyce Odeke Akello, Institute for Infectious Diseases, University of Bern, Friedbühlstrasse 51, 3001 Bern, Switzerland. Phone +41316328646, Email: joyce.akello@ifik.unibe.ch

Materials

Environmental samples

- Samples (ticks, mosquitoes)

Reagents

- QIAzol lysis reagent (Qiagen, Cat No./ID: 79306). **IMPORTANT** QIAzol contains phenol and high amount of chaotropic salts. See safety data sheets for further information.
- PBS
- Proteinase K (ThermoFisher, Cat. No. 25530049)
- Chloroform
- Silica magnetic beads (G-Biosciences; VWR International, cat.no.786-915). **IMPORTANT** use of other Silica magnetic beads may affect the yield of recovered nucleic acid.
- Isopropanol
- RWT buffer (Qiagen, Cat. No 1067933). **IMPORTANT** RWT buffer contains some amount of guanidinium thiocyanate. See safety data sheets for further information. **CRITICAL** requires addition of ethanol by the user just before using for the first time.
- RPE buffer (Qiagen, Cat. No 1018013). **CRITICAL** requires addition of ethanol by the user just before using for the first time.
- Nuclease free water (ThermoFisher, Cat. No R0581)
- TE Buffer (0.1mM) (Invitrogen, Cat. No 12090-0159
- SuperScript® IV First-Strand Synthesis System (ThermoFisher, Cat. No. 18091050)
- Ethanol absolute (Merck Cat. No 1.00983.1000)
- Qubit dsDNA HS Assay Kit 100 0.2-100ng (Invitrogen, Cat. No Q32851)
- QIAquick PCR Purification Kit (Qiagen, Cat. No 28106)

- AFA grade Water (Covaris, Cat. No R000161)
- AMPure XP 60mL (Beckman Coulter, Cat. No A63881)
- High Sensitivity DNA kit (Agilent Technologies, Cat. No 5067-4626)
- Ion Plus Fragment Library Kit (Life Technologies, Cat. No 4477597)
- Ion Plus Fragment Library Adapter (Life Technologies, Cat. No 4476340)
- Ion Xpress ™ Barcode Adapters 1-16 Kit, P1 adapter (Life Technologies, Cat. No 4471250)
- Ion Xpress ™ Barcode Adapters 17-32 Kit, P1 adapter (Life Technologies, Cat. No 4474009)
- Ion 520™ & Ion 530™ Kit-Chef (Life Technologies, Cat. No A30010)
- Ion S5™ Calibration Standard (Life Technologies, Cat. No A27988)
- Ion 520™ Chip Kit (Life Technologies, Cat. No A27762)
- Ion 530™ Chip Kit (Life Technologies, Cat. No A277649
- Ion Sphere™ Quality Control Kit (Life Technologies, Cat. No 4468656)
-

Equipments

- Gloves (textured single-use nitrile gloves; Ansell Health Care, cat. no. 588783; and Rotiprotect-latex gloves, type 2, powder-free; Roth, cat. no. L950.1)
- Centrifuge
- TissueLyser (Qiagen) or Precellys 24 (Labgene Scientific)
- Thermomixer
- Magnetic rack
- 1.5 mL DNA LoBind Tube (Eppendorf Cat. No 022431021)
- 1.5 mL Safe Lock Tubes PCR clean (Eppendorf Cat. No 0030123.328)
- 2.0 mL DNA LoBind Tube (Eppendorf Cat. No 022431048)
- 0.2 mL Micro Amp 8-Tube Strip (Applied Biosystems Cat. No N8010580)
- Ice bucket
- 0.5-10µL pipette (Eppendorf)
- 10-100µL Pipette (Eppendorf)
- 50-200µL pipette (Eppendorf)
- 100-1000µL pipette (Eppendorf)
- Vortex VWR VV3, MiniStar, PCR Strips VWR
- Thermomixer comfort (Eppendorf)
- PCR Hood Captair (Bio by Erlab)
- Thermal Cycler machine (AB Applied Biosystems)
- Qubit 2.0 Fluorometer (Invitrogen)
- Centrifuge 5810R (Eppendorf)
- Vacuum pump
- Milli-Q System Milli-Q
- 0.2mL Qubit Assay Tubes (Life Technologies Cat. No Q32856)
- Ultrasonicator M220 Covaris
- MicroTube insert 500301 Covaris
- MicroTube AFA Fiber Screw Cap (Covaris Cat. No 520096)
- M220 Swab Cleaning Kit (Covaris Cat. No 500298)
- Library Builder (Life Technologies)
- DYNAL Magnetic rack (Invitrogen)
- Bioanalyzer 2100 (including Chip Priming Station, IKA Vortexer Model MS3 with chip adapter, Agilent Technologies, Cat. No 5065-4401)
- Ion Chef Instrument Life Technologies

A.) Protocol for extraction method 5 - ROVIV (Recovery of Viruses in Vectors). * Timing approximately 50 mins per 2 samples

Note: Extraction is performed after homogenization of ticks and or mosquitoes using the TissueLyser (Qiagen) or Precellys 24 sample homogenizer (Labgene Scientific)

__Lysis__

1. Add **1000 µl** of QIAzol reagent to **200 µl** of homogenized supernatant sample
2. Pipet the sample up and down several times to homogenize
3. Add **50 µL of Proteinase K** to the mix. Mix thoroughly by vortexing or pipetting. Incubate the sample for 15 min at 56° C in a thermomixer.
4. Add **200 µl chloroform** to each sample.
5. Mix by inverting the tube vigorously and incubate for 5 min at room temperature
6. Centrifuge at 12,000 g for 10 min at 4^0 C. The mixture separates into the lower red phenol-chloroform, and interphase, and a colorless upper aqueous phase
7. Transfer the upper aqueous phase (**600 µl**) containing the RNA to new 2.0 ml labelled tube
8. Add **25 µl of silica magnetic beads (G-Biosciences)** resuspended well by vortexing **and 450 µl of Isopropanol** (100%)
9. Mix briefly by vortexing
10. Briefly centrifuge for 5 sec and place on magnetic rack for 2 min
11. Take off the supernatant

__Washing the Silica-pellet__

12. Remove the sample from the magnet and add **700 µl buffer RWT**
13. Resuspend the magnetic beads by vortexing
14. Place the tube on the magnetic rack and let the magnetic beads collect on the magnet for 2 min.
15. Take off the supernatant
16. Remove the sample from the magnet and add **500 µl buffer RPE**
17. Resuspend the magnetic beads by vortexing
18. Place the tube on the magnetic rack and let the magnetic beads collect on the magnet for 2 min.
19. Take off the supernatant
20. **Repeat step 16 -19**
21. Make sure that all the supernatant is removed completely (use a small volume pipette tip and make sure no droplets are left on the tube wall).
22. Dry the silica for 5 min at room temperature (15-25° C). The sample tube must be left on the magnetic rack as the silica dries

__Elution of nucleic acids__

23. Remove the sample tube from the magnetic rack and add **50 µl** nuclease free water.
24. Resuspend the magnetic beads by vortexing
25. Incubate **for 5 min at 56° C** in thermomixer at **300 rpm**, with continuous shaking.
26. Centrifuge briefly and place the tube on the magnetic rack
27. Let the magnetic beads collect at the magnet for 2 min.
28. Carefully transfer **50 µl** supernatant containing viral RNA to a new, RNase free tube.
29. Use the purified RNA immediately in downstream applications or store at -80° C until use. Note: Keep RNA samples on ice after extraction and while working with it.

B.) cDNA synthesis. * Timing approximately 35 min

30. Freeze thaw the random hexamers (50ng / µl), dNTP mix, 5x SSIV buffer and 100 mM DTT on ice

31. Preheat the thermal cycler to 75 ° C

preparation of master mix I

Master mix I

**component**	**Volume per reaction**
Random hexamers (50 ng/µl)	1 µl
10 mM dNTP mix (10 mM each)	1 µl
Template RNA	11 µl
**Total volume**	**13 µl**

32. mix the contents and briefly centrifuge.
33. Incubate for 5 min at 75° C in a thermal cycler with hot lid on (105° C) and then immediately snap cool the reaction for at least 1 min on ice.
34. Briefly Centrifuge the PCR Strip.
35. Vortex and briefly centrifuge the 5x SSIV buffer.
36. Combine the following components in a PCR reaction tube.

Master mix II

**component**	**volume per reaction**
5x SSIV buffer	4 µl
100 mM DTT	1 µl
Ribonuclease inhibitor	1 µl
Superscript IV reverse transcriptase (200 U / µl)	1 µl
**Total volume**	**7 µl**

37. Mix and briefly centrifuge contents.
38. Add the reaction master mix II to the annealed RNA
39. Incubate the combined reaction mixture in the thermal cycler with hot lid on (105° C) as follows:
    23° C for 10 min
    55° C for 10 min
    80° C for 10 min
    4° C ∞

***Pause point.** Store the cDNA at -20° C for future use or proceed directly to use for next step,

C. cDNA quantification. *Timing approximately 35 min including pre setup and running time.

Measure DNA with Qubit (Range 10pg / µl-100ng / µl) using the Qubit dsDNA HS Assay Kit.

Set up

40. Bring standard 1 and standard 2 to room temperature for 20 minutes before use
41. Prepare working solution: dsDNA HS Reagent 1: 200 dilute
42. For each measurement, the final volume must be 200µl

CAUTION: Use the 0.2ml Qubit assay tubes for DNA measurements in the Qubit. The Qubit assay tubes are very thin-walled and can get small cracks quickly, so it is recommended to close and open the tubes in a rack.

43. Pipette 190 µl Working Solution into the two standard tubes.
44. Pipette 10 µl from standard 1 or standard 2 into the appropriate tube (The fluorometer must be calibrated once a day).
45. vortex the tubes and briefly centrifuge.
46. Incubate the tubes at room temperature for 2 min.
47. Measure the tubes in the fluorometer as follows:
    - Press "DNA" on the home screen
    - Select "dsDNA High Sensitivity"
    - Press "Yes" on the standard screen
    - Place standard 1 in the fluorometer
    - Press "read"
    - Place standard 2 in the fluorometer
    - Press "read"

Measuring samples

48. Pipette 198 µl of working solution in each qubit assay tube
49. Pipette 2 µl of sample into the assay tube containing 198 µl of working solution
50. Vortex and briefly centrifuge.
51. Incubate at room temperature for 2 min.
52. Place the tube in the fluorometer
53. Press "read next sample" key
54. To calculate the concentration of the original sample, press "Calculate Stock Conc."
55. Select the volume used, the measuring sample, in the drop-down list (1-20 µl).
56. Select the concentration as ng / ul

D. cDNA fragmentation. *Timing approximately 35 min including pre setup and running time.

CAUTION: For the best SeqPlex amplification genome coverage and NGS results, the DNA to be sequenced must be fragmented in approximately 200bp-500bp DNA fragments. In addition, the Ion Torrent platform sequences DNA fragments around the 400bp without adapters (adapter approx. 70bp) and barcode (approx. 85bp).

57. Dilute DNA to the concentration as required for the SeqPlex amplification. The amount of DNA for the seqplex amplification should be up to 1ng per reaction.
58. Pipette 50 µl sample into the Covaris micro tube (if the sample volume is less than 50 µl, fill up to 50 µl with nuclease free water).

Note: Keep diluted DNA in the refrigerator until Covaris is ready.

Optional: Place 1µL of diluted DNA into a LoBind tube in the refrigerator for later analysis on the bioanalyzer.

59. Turn on Covaris about 30 min before use and fill with AFA water until the Covaris software gives green light for the water level.

CAUTION: Water temperature must be 20 ° C

60. Transfer samples to a micro tube screw-cap.
61. Screw the Micro tube screw-cap into the water bath.
62. Run the program for 400bp sequencing as follows:

Time	60 sec
Temp.	20 ° C
Duty Factor	20
Cycles / burst	200
Peak Power	50

63. Centrifuge the micro tube screw-cap briefly.
64. Pipette the fragmented sample from the micro tube screw-cap into a 1.5mL LoBind tube
    Optional: Place 1μL of the sample in a LoBind tube in the refrigerator for later analysis on the Bioanalyzer
65. Store the sample in the refrigerator until further processing

E. Double stranded DNA synthesis – (SeqPlex WGA). *Timing approximately 5 hours and 30 min

Note: For best results, use up to 1 ng per reaction of fragmented sample

Amplification with the SeqPlex WGA kit.

66. Thaw the Library Preparation Buffer (LP100), 5x Amplification Buffer (A5112) and water (W4502). Mix thoroughly
67. Combine the following component in PCR reaction tube

Component	Volume per reaction
LP100	2 μl
Fragmented DNA	X μl (up to 1 ng)
Water (W4502)	Y μl
**Total volume**	**14 μl**

68. Mix thoroughly, centrifuge briefly, and incubate in a thermal cycler for 2 min at 95 °C, followed by cooling on ice for 2 min
69. Add 1 μL of Library Preparation Enzyme (E0531), cap tube and mix thoroughly.
70. Centrifuge briefly and immediately place reaction in a thermal cycler incubating as follows:
    16 °C for 20 min
    24 °C for 20 min
    37 °C for 20 min
    75 °C for 5 min
    4 °C ∞
71. Remove sample from thermal cycler and briefly centrifuge.

***Pause point.** Amplification may be completed immediately or store Pre-amplification product at -20° C for up to three days.

72. Add the following reagents to the 15 μl of Pre-amplification product from step 71. (For multiple reactions, a master mix may be prepared. Add 60 μl of the master mix to each reaction):

Component	Volume per reaction
5X Amplification mix (A5112)	15 μl
DNA Polymerase for SeqPlex (SP300)	1.5 μl
Pre-amplification product	15 μl
Water (W4502)	43.5 μl
**Total reaction volume**	**75 μl**

73. Mix thoroughly and incubate in the thermal cycler as follows:
    94 °C for 2 min Initial Denaturation
    94 °C for 15 sec Denature
    70 °C for 5 min Anneal / Extend
    } 24 Cycles

70 °C for 30 min
4 °C ∞

CAUTION: The extended incubation at 70° C for 30 min is absolutely essential for efficient primer removal.

***Pause point.** Reactions may be purified immediately or stored at –20° C until purification

74. Purify using QIAquick PCR purification Kit (Qiagen, Cat. No 28106) as follows: elute in 50 µL TE buffer.

    i. Using the pipette, measure the volume of the PCR product and pipette into a 1.5mL Eppendorf LoBind tube.
    ii. Add 5 times the volume of PB buffer to the measured PCR product volume and mix well
    iii. Transfer the violet column to a 2 ml collection vessel
    iv. Pipette the entire contents of the tube into the violet column
    v. Centrifuge the column at 17`900 x g for 60 sec
    vi. Discard liquid in the 2 ml collecting vessel
    vii. Place the column in the same collecting vessel
    viii. Pipette 750 µL PE buffer into the column
    ix. Centrifuge the column at 17`900 x g for 60 sec
    x. Discard liquid in the 2 ml collecting vessel
    xi. Place the column in the same collecting vessel
    xii. Centrifuge the column at 17`900 x g for 60 sec
    xiii. Transfer the column to a 1.5ml LoBind tube
    xiv. Pipette 30µL-50µL TE buffer directly onto the membrane without touching the membrane
    xv. Incubate for 1 min at room temperature
    xvi. Centrifuge the column at 17`900 x g for 60 sec
    xvii. In the 1.5ml LoBind tube is the purified DNA

    ***Pause point.** DNA can be further processed or stored at 4 ° C / -20 ° C

75. Determine the purified DNA's concentration by using Qubit (step 40 -56). The DNA yield at this point will vary depending on the quality of starting DNA, but is usually 1-5 µg.

Primer removal

Note: A reaction input of 2.1 µg is recommended to yield sufficient product for subsequent deep sequencing workflows (>1 µg).
Determine the purified DNA's concentration by using Qubit (step 40 -56). The DNA yield at this point will vary depending on the quality of starting DNA, but is usually 1-5 µg.

76. Combine the following component on ice:

Component	Volume per reaction	
10X Primer removal buffer (SR401)	8.0 µl	
Primer removal solution (SR400)	1.6 µl	
2.1 µg of purified SeqPlex DNA	X µl	
Water (W4502)	Y µl	
Primer removal enyzme (SR402)	3.75 µl	
Total reaction volume	75 µl	76

77. Mix thoroughly, briefly centrifuge and incubate the total reaction mix in a thermal cycler as follows:

    37 °C for 60 min
    65 °C for 20 min
    4 °C ∞

78. Remove reactions from the thermal cycler, and centrifuge briefly.

*__Pause point.__ Reactions may be purified immediately or stored at –20° C for up to three days.

79. Purify the remaining reaction of primer removal enzyme using the QIAquick PCR purification Kit as described previously in step 74.

F. Automated Library preparation using library builder for sequencing with the Ion Torrent S5 platform. *Timing approximately 3 hours and 30 min (including pre setup and running time),

80. Thaw the Ion Plus Fragment Library Kit Cartridges and LdN Buffer (45 min at room temperature at maximum 2 hours). If the LdN buffer has white precipitate, heat the tube at 37 ° C in the thermal block, place the Ion End Repair Enzyme Mix in the refrigerator, bring E1 buffer and Agencourt AMPure XP beads to room temperature.
81. Freeze thaw the adapter and / or barcodes on ice, vortex and centrifuge briefly.
82. Insert the "Ion Plus and Ion Xpress Plus" program card into the Library Builder. **CRITICAL** Only when the Library Builder is switched off. Activate the library builder.

    Note: The library is ligated with adapters A and P1 so that the sequencing can take place. Furthermore, you have the option to load your library with a barcode (Adapter Barcode and P1 Adapter) to load several libraries on one chip. Use the tables below to select the volume of adapters or barcodes.

Library without Barcode (Ion Plus Fragment Library Adapters)										
**Input**	**50-100 ng**	**100-200 ng**	**200-300 ng**	**300-400 ng**	**400-500 ng**	**500-600 ng**	**600-700 ng**	**700-800 ng**	**800-900 ng**	**900-1000 ng**
Adapter	1	2	3	4	5	6	7	8	9	10
E1 buffer	49	48	47	46	45	44	43	42	41	40
**Total Volume**	**50**	**50**	**50**	**50**	**50**	**50**	**50**	**50**	**50**	**50**

Library with Barcode (Ion Plus Fragment Library Adapters)										
**Input**	**50-100 ng**	**100-200 ng**	**200-300 ng**	**300-400 ng**	**400-500 ng**	**500-600 ng**	**600-700 ng**	**700-800 ng**	**800-900 ng**	**900-1000 ng**
Ion Xpress p1 Adapter	1	2	3	4	5	6	7	8	9	10
Ion Xpress Barcode X	1	2	3	4	5	6	7	8	9	10
E1 Buffer	48	46	44	42	40	38	36	34	32	30
**Total volume**	**50**	**50**	**50**	**50**	**50**	**50**	**50**	**50**	**50**	**50**

83. Mix the diluted adapters (50 µl) with the LdN buffer by pipetting up and down then centrifuge the tube briefly
84. When the cartridges have completely thawed, make sure that all the solutions are at the bottom of the wells by gently tapping on the bench top
85. Loading and programming the Library Builder: set up following the manufacturer's instructions.

86. Select the program on the Library Builder as follows:
    - No size selection
    - Pre-sheared
87. Once the programme is complete, remove the library from the machine and transfer it to a new clean 1.5 ml LoBind tube.

***Pause point.** Store the library in the refrigerator for up to 1 week or freeze in a freezer at -20° C for longer storage

88. Optional: use 1µl of the library for analysis on the bioanalyzer

G. Size selection of library and clean up. * Timing approximately 35 mins

89. Calculating Agencourt AMPure XP beads set for the Size Selection. 0.55 x Volume of library = Agencourt AMPure XP beads (Size Selection above 400bp (> 400bp)). Calculation example: Measure the volume of the library e.g 28 µl library x 0.55 = 15.4 µl Agencourt AMPure XP beads.
90. Vortex the Agencourt AMPure XP beads well, add the calculated amount of Agencourt AMPure XP beads into the library, and mix by flicking the tube
91. Incubate the mixture for 3 mins at room temperature on shaker at 300 rpm
92. Transfer the tube mixture on the magnetic rack and incubate for 3 mins at room temperature to separate beads from the supernatant.
93. Carefully pipette the clear solution (supernatant) from the tube mixture without disturbing the beads into a new LoBind tube. **Note**: This is the size selected library.
94. Calculating amount of Agencourt AMPure XP beads for clean up. Measure the volume of the size selected library, and multiple this volume by 1.8. this will give the volume of AMPure beads required for clean up.
95. Add volume of AMPure beads required for clean up to the tube containing the size selected library
96. Incubate for 5 min at room temperature on a shaker at 300 rpm
97. Place the sample tube on the magnetic rack and incubate for 3 min at room temperature (until a clear supernatant solution forms and beads have concentrated on the wall of the tube).
98. Whilst the tube is still on the magnetic rack, carefully pipette and discard the supernatant.
99. Add 200 µl of 70% ethanol into the sample tube without disturbing the bead pellet and incubate for 30 sec at room temperature.
100. Carefully pipette the ethanol from the sample tube and discard.
101. Repeat step 105 -106 making sure that the sample tube is still on the magnetic rack
102. Allow the pellet to air dry for 5 mins at room temperature. Make sure not to over dry the pellet.
103. Remove the sample tube from the magnetic rack and resuspend the pellet with 30 µl of TE buffer (pipette at least 10 times with the pipette tip up and down)
104. Incubate for 2 min at room temperature
105. Place the sample tube on the magnetic rack, incubate for 2 min at room temperature and pipette the clear supernatant (eluate = DNA library) into a new LoBind tube.

***Pause point.** Store the DNA library at 4° C for up to 1 week or freeze in a freezer at -20° C for longer storage or continue directly with the quality check.

H. Quantification and quality check of the DNA library. * Timing approximately 2 hours and 30 min including set up and running time

106. Measure the concentration of the DNA library using Qubit (step 40 -56) and dilute the library to be analyzed by the bioanalyzer to 1-10 ng / µl in nuclease free water.
107. Check for the quality of the DNA library using an Agilent Bioanalyzer High Sensitivity DNA assay as described in the manufacture's manual. Note: this is to ensure that the fragment length of the DNA library is within the expected range (200 - 400bp) for sequencing on the Ion torrent S5 platform.
108. Quantification of the DNA library. Calculate the molar concentration in pM of the library by creating an excel table of the Ion Torrent "Library Conversion Table (pg / uL to pM)" using the formula below;

$$\text{Molar concentration in pM} = \frac{1}{(660 \text{ X Peak size of library}) \text{ X } (\text{Stock library in pg/ }\mu\text{l}) \text{ X } 1000000}$$

Example:

- Concentration of sample determined by Qubit = 9.12ng/µl
- Stock library in pg/µl = 9.12ng/ul x 1000 = 9120pg/µl
- Peak size (bp) of the library determined by the Bioanalyzer = 300

$$\text{Therefore, Molar concentration} = \frac{1}{(660 \text{ X } 300) \text{ X } (9120) \text{ X } 1000000}$$

Molar concentration = **46060.6pM**

I. Deep sequencing. * Timing approximately 23 hours including pre setup and running time.

109. Calculating the estimated dilution factor for the DNA library. Divide the calculated pM concentration from the library conversion excel table by 3 (e.g. 38071 / 3 = 12690.3pM), then further divide this value by 80 (e.g 12690.3 / 80 = 158.6). This gives the dilution factor of 1: 159 for the DNA library. Note: we recommend diluting the DNA library in E1 or TE buffer. In-house laboratory optimization (data not shown) indicated that estimating the dilution factor of the DNA library using this formula yields enough amount of library required for loading on the Ion torrent chip for sequencing on the Ion S5 platform.
110. Automated loading of library on the chip using Ion Chef system. As described in the manufacture's manual and protocols,
111. Sequencing with Ion torrent S5 platform. As described in the manufacture's manual and protocols.

Supplementary file 2

Evaluation of Viral RNA Recovery Methods in Vectors by Metagenomic Sequencing.

Joyce Odeke Akello[1, 2, 3*], Stephen L. Leib[2], Olivier Engler[1], Christian Beuret[1*]
[1]Biology Division, Spiez Laboratory, Swiss Federal Office for Civil Protection, Austrasse, CH-3700 Spiez, Switzerland.
[2]Institute for Infectious Diseases, University of Bern, Friedbühlstrasse 51, 3001 Bern, Switzerland.
[3]Graduate School for Cellular and Biomedical Sciences, University of Bern, Hochschulstrasse 4, 3012 Bern, Switzerland.

[*]**Correspondence**

Dr. Christian Beuret, Spiez Laboratory, Swiss Federal Office for Civil Protection, Austrasse CH-3700, Spiez, Switzerland. Phone +41584681664, E-mail: Christian.Beuret@babs.admin.ch

Joyce Odeke Akello, Institute for Infectious Diseases, University of Bern, Friedbühlstrasse 51, 3001 Bern, Switzerland. Phone +41316328646, Email: joyce.akello@ifik.unibe.ch

Table S1. Primers and probes used for detection in the real time PCR assay

**Tick borne encephalitis virus**		**Sequence 5' - 3'**	**Gene**
TBEEF6	Forward primer	GGCTTGTGAGGCAAAAAAGAA	E gene
TBEER2	Reverse primer	TCCCGTGTGTGGTTCGACTT	
TBEEP4	TaqMan probe (FAM, BHQ-1)	AAGCCACAGGACATGTGTACGACGCC	
**Dengue virus**			
DENV-2Fmod15	Forward primer	CAGGYTATGGCACHGTYACGAT	E gene
DENV-2Rmod15	Reverse primer	CCATYTGCAGCARCACCATCTC	
DENV-2 Pmod15	TaqMan probe WT (HEX, BHQ-1) (FAM, BHQ-1)	CTCYCCGAGAACRGGCCTCGACTTCAA	
**Zika virus**			
ZIKV_NS3_MGB_F3	Forward Primer	TGCACACTGGCTTGAAGCA	NS3
ZIKV_NS3_MGB_R3	Reverse Primer	AGCTTRAACTCTCCCTCAATGG	
ZIKV_NS3_MGB_P3	Probe (MGB)	TGGCCTCATAGCCTC	
**Chikungunya virus**			
CHIKIOS	Forward primer	CCGACTCAACCATCCTGGAT	NSP
CHIKIOAs	Reverse primer	GGCAGACGCAGTGGTACTTCCT	
CHIKIOP	TaqMan probe WT (FAM)	TCCGACATCATCCTCCTTGCTGGC	
**Langat virus**			
LGTV F	Forward primer	TGTGTGGAGCGGCGATT	NS3
LGTVR	Reverse primer	TAAGGGCGCGTTCCATCTC	
LGTVP	TaqMan probe (FAM, BHQ-1)	CTTGGCCCCCACACGAGTGGTG	
